# Removal of Lead, Cadmium, and Mercury in Monometallic and Trimetallic Aqueous Systems Using *Chenopodium album* L.

**DOI:** 10.1155/sci5/6842159

**Published:** 2024-12-11

**Authors:** Susan S. Flores-Calla, José A. Villanueva-Salas, Karla Diaz-Rodriguez, Elvis G. Gonzales-Condori

**Affiliations:** ^1^Escuela de Postgrado, Universidad Católica de Santa María, Urb. San José s/n Umacollo, Arequipa, Peru; ^2^Grupo de Investigación en Biotecnología y Ciencia de Los Alimentos, Universidad Tecnológica del Perú, Av. Tacna y Arica 160, Arequipa, Peru

## Abstract

The presence of heavy metals in water represents a risk to the life of all species on the planet. Phytoremediation is an effective alternative to remove heavy metals from contaminated aqueous environments. In the present research, *Chenopodium album* L. was examined for the remediation of waters contaminated with Cd, Pb, and Hg. Studies were carried out in waters containing each metal separately (monometallic aqueous systems) and in mixtures (trimetallic aqueous systems). First, the adaptation of *Chenopodium album* to different concentrations of Hoagland's nutrient solution (HNS) was evaluated, then, a phytotoxicity study was carried out to determine the appropriate concentrations of each metal to test the tolerance of the plant during the accumulation study, and finally, the bioaccumulation capacity of *Chenopodium album* for Cd, Pb, and Hg was evaluated. *Chenopodium album* showed tolerance to levels of 5 mg/L Hg and 10 mg/L Cd and Pb in 25% HNS. The bioaccumulation tests showed that *Chenopodium album* can remediate Cd, Pb, and Hg contaminated waters in both monometallic and trimetallic aqueous systems. These findings suggest important future applications in the food industry for the production of *Chenopodium album* as we demonstrate that this species adapts and grows in hydroponic media. In particular, the ability of *Chenopodium album* to adapt to extreme conditions could be exploited for further studies on phytoremediation of heavy metals in river water, irrigation water, wastewater, effluents, and mine tailings.

## 1. Introduction

Water is the most important compound and one of the main constituents of nature and living beings [1]. Water pollution is one of the biggest problems for growing human population worldwide today [2, 3]. This is because global development in recent decades has been characterized by a rapid increase in the use of chemicals associated with different types of activities: industrial, urban, commercial, and agricultural [4, 5]. The main sources of water pollution are industrial activities, which generate different toxic products as a result of poor waste management, ending up in rivers, lakes, and aquifers, which alter the natural quality of water, putting the water supply at high risk [6].

One of the most serious environmental problems is water contamination by heavy metals, which are toxic and capable of altering the metabolism of living beings, causing serious teratogenic and carcinogenic disorders [7]. Among the heavy metals present in contaminated water cadmium (Cd), lead (P), and mercury (Hg) are highly toxic [8].

Cd is distributed in deposited, dissolved, adsorbed, or bioaccumulated forms in aquatic ecosystems [9]. Ecological balance may be disturbed by the presence of Cd [10]. Cd is a toxic heavy metal and its chronic exposure leads to alterations in living organisms when it enters the food chain [11]. The greatest health threats of chronic exposure to Cd through air or food are renal tubular dysfunction, followed by bone degeneration related to alterations in calcium metabolism, osteoporosis, and osteomalacia [12]. Pb is also dangerous for the environment and its effects in humans are devastating [13]. There is virtually no function in the human body that is not affected by Pb toxicity. This metal is very persistent in the environment and, due to its continued use; its levels are increasing in almost all countries, posing a serious threat to the environment [14]. In humans, toxic effects of Pb on bone, renal, reproductive, and nervous systems have been reported [15, 16]. On the other hand, Hg plays a key role in serious health problems due to environmental or occupational exposure [17]. Aquatic ecosystems are an essential component of the global biogeochemical cycling of Hg, as in these environments inorganic Hg can be converted to toxic methylmercury, and reemissions of elemental Hg rival anthropogenic Hg emissions on a global scale [18, 19]. Elemental Hg is absorbed mainly by inhalation. Absorption by ingestion is minimal and it does cross the blood-brain barrier, where it can be deposited [20]. Elemental Hg is more volatile when heated and is readily absorbed when aerosolized by suction [21]. Pulmonary, central nervous system, and renal toxicity are common with elemental Hg [22]. Inorganic Hg salts are mainly absorbed from the gastrointestinal tract and secondarily through the intact skin [23] and the salts cause mainly renal and gastrointestinal toxicity [24]. Organic Hg toxicity often causes neurological symptoms [23].

Heavy metals also negatively affect water resources. Fish are highly susceptible to the toxic effects of Pb through bioaccumulation in specific tissues [25] causing oxidative stress by excessive production of reactive oxygen species (ROS), as well as inducing neurotoxicity and influencing immune responses [26]. On the other hand, Cd bioaccumulates and is distributed in tissues, damaging the integrity of tissue structure and function [27]. Cd exposure can also have a fatal effect through oxidative stress and immunotoxicity in aquatic organisms [28]. Also, Hg can bioaccumulate in fish and can produce teratogenic, neurotoxic, and reproductive toxicity effects causing damage to cells, tissues, proteins and genes, jeopardizing the survival, growth and behavior of marine fish [29].

Despite the obvious toxicity of these heavy metals, their use continues to be common throughout the world due to their multiple applications, so it is important to develop processes that allow the treatment or removal of these heavy metals from contaminated water bodies [30]. There are several physicochemical methods for the removal of heavy metals from water; however, most of them are expensive and not very feasible at the industrial level [31]. Currently, the development of new technologies that are more feasible and compatible with the environment is under investigation, such as the case of phytoremediation [32].

Phytoremediation proposes an in situ treatment of contaminated areas, whether water, air, or soil, using plants, which can reduce the toxic effects of the pollutant, through degradation, assimilation, or metabolism of organic and inorganic agents in the environment [33]. It is an emerging technology with great potential for efficient cleaning, as it is more sensitive to environmental stress, easy to handle, and low cost [34].

Among the plants used in heavy metal phytoremediation, the *Chenopodium* genus has a history of tolerance to heavy metals during its growth, adapting to contaminated environments, which allows the accumulation of these contaminants in the plant [35–39]. *Chenopodium album* L. (*C. album*) is popularly known as Liccha in Perú. It is a plant considered a weed, with abundant and fast growth, in different agricultural areas of the city of Arequipa, Peru. Several studies have investigated the accumulation capacity of *C. album* under various conditions since, this plant has demonstrated a capacity for colonization in contaminated areas [40], and has been proven to remove Cd [41, 42], Pb [43], Cu, Zn, Hg, Ni [44] Mn, Sr, Ba, He [45], ^133^Cs [46], and Cr (VI) [47]. In another phytoremediation study on soils simultaneously contaminated with Cd, As, Pb, Cu, Zn, and Cr, it was shown that *C. album* could accumulate only one metal [48]. Laboratory scale studies were also developed in MS medium, tolerating concentrations of up to 50 mg/L of Pb [49]. On the other hand, it was demonstrated that this plant species could grow, adapt and accumulate heavy metals present in paper mill wastewater [50], sewage sludge [51]. With this background *C. album* would be a potential candidate species for application in phytoremediation in any growth medium [51], due to its ability to adapt to and withstand extreme environmental conditions [52]. However, its phytoremediation capacity in hydroponic systems is not known yet, so this research would be the first study to demonstrate that *C. album* can also adapt to hydroponic media contaminated with heavy metals, which could be used for the remediation of river, lake and tailings waters with high heavy metal loads.

Therefore, the objective of the present research was to evaluate *C. album* as a potential phytoremediator of Cd, Pb, and Hg in water systems. The experiments were carried out in waters contaminated with each metal separately (monometallic aqueous systems) and the phytoremediation capacity of *C*. *album* was studied in the presence of the three metals in mixture (trimetallic aqueous systems), since current studies in most cases only study the remediation of metals in monometallic aqueous systems.

## 2. Materials and Methods

### 2.1. Reagents and Equipment

All reagents and standards were obtained from Merck. Suprapure nitric acid was used. Hg chloride (HgCl_2_), Cd nitrate tetrahydrate [Cd(NO_3_)_2_·4H_2_O], and Pb nitrate [Pb(NO_3_)_2_] were analytical grades. To validate the method for the quantification of Cd, Pb, and Hg, standards obtained from Merck of 1000 mg/L of each metal were used. Hoagland nutrient solution (HNS) consisting of solution A and solution B was purchased from the Universidad Agraria La Molina in Peru. Solution A consists of potassium nitrate 110 g/L, ammonium nitrate 70 g/L triple superphosphate 36 g/L. Solution B consists of magnesium sulfate 110 g/L, iron chelate 6% 8.5 g/L, and micronutrient solution 20% v/v. The micronutrient solution consists of manganese sulfate 5 g/L, boric acid 3 g/L, zinc sulfate 1.7 g/L, copper sulfate 1 g/L, and ammonium molybdate 0.2 g/L.

Ultrapure water (18.2 M′Ω) was obtained from the Simplicity System Merck Millipore water purifier. Digestion of the plant material was carried out in the MARS6 230/60 CEM One Touch microwave digester. Post-microwave digestion was performed on the Metrohm 705 UV Digester Digestion System. Metal analyses were performed on a Perkin Elmer 5300 inductively coupled plasma-optical emission spectrophotometer (ICP-OES).

### 2.2. Collection and Identification of Plant Material

The plant species for the study were collected from the fields of Cayma, Arequipa, Peru (−16.3423329, −71.5386266). Stem fragments were cut from healthy, young plants and used for propagation. The plant species was identified as *Chenopodium album* L. (*C. album*) in the “Herbarium Areqvipense” (HUSA) of the Universidad Nacional de San Agustín de Arequipa, Peru.

### 2.3. Propagation Test

Vegetative propagation by cuttings was carried out. For this purpose, the healthiest plants were selected and washed with 5% sodium hypochlorite to eliminate bacteria and fungi that were impregnated. Then, stem fragments (cuttings) were cut and planted in trays with sterile humid soil for propagation for 20 days, until the seedling was fully developed.

### 2.4. Adaptation of *Chenopodium album* L.

HNS was used, which was prepared by mixing 5 mL of solution A and 2 mL of solution B in 1 L of water; this solution was considered 100%. Three treatments were prepared using HNS concentrations of 25% (T1), 50% (T2), and 100% (T3). Then, for each treatment, 5 seedlings were placed in submerged trays for 15 days to determine the HNS concentration that allows the best development of *C. album*. The best treatment corresponds to the one that allows greater growth of *C. album*. Once the best HNS concentration was defined, more seedlings were adapted for an adaptation time of 20 days for subsequent experiments.

### 2.5. Phytotoxicity Test

The phytotoxicity test in water consisted of preparing treatments with concentrations of Cd, Pb, and Hg diluted in 25% of HNS. Concentrations of 0, 1, 5, and 10 mg/L for Hg were prepared using HgCl_2_, and 0, 5, 10, and 15 mg/L for Pb and Cd using Pb(NO_3_)_2_ and Cd(NO_3_)_2_, respectively. These concentrations were chosen based on preliminary experiments. Once the contaminated solutions were prepared, three seedlings of *C. album* were placed in contact with experimental solution by immobilizing the stems of the seedlings in a hole of a polystyrene sheet, leaving the roots submerged in the contaminated solutions. The phytotoxicity test lasted 19 days because the longer the number of days, no differences was found in the toxicity characteristics of the seedlings. The evaluations were performed on days 0, 1, 4, 7, 10, 13, 16, and 19. All assays were performed in triplicate. To determine the degree of toxicity, the toxicity levels proposed by the Expert Committee on Plant Health were used [53], for this purpose, periodic observations were made and the toxicity grades were classified as shown in Table 1.

### 2.6. Bioaccumulation Assay

The bioaccumulation assay was carried out in 12 × 25 × 30 cm glass containers in which Cd, Pb, and Hg solutions were placed in monometallic and trimetallic aqueous systems at tolerable concentrations for *C. album* according to results of the phytotoxicity test. Diffuser tubes connected to peristaltic pumps were placed at the bottom of each vessel to bubble air and keep each system in constant motion. Finally, polystyrene trays were submerged with ten *C. album* seedlings for each container. Ten *C. album* seedlings were used in 3 L of contaminated hydroponic solution prepared in the laboratory. All experiments were performed in triplicate.

One plant was randomly sampled every 5 days for 30 days. No replenishment of metal concentrations was performed during the 30 days. For the quantification of Cd, Pb, and Hg, each plant sampled was first washed with abundant distilled water and then placed on Kraft paper and dried under shade at room temperature [54]. Subsequently, the plants were sectioned into aerial and root parts, and then chopped with the help of a scalpel and pulverized in an agate mortar. 100 mg of the pulverized aerial and root parts were weighed and placed in the digestion tubes, then, 2 mL of supra pure HNO_3_ was added and finally, it was taken to the MARS 6 microwave digester. The digested solution was then placed in a 25 mL flask and made up to volume with ultrapure water. Subsequently, the concentration of Cd, Pb, and Hg was determined by ICP (ICP-OES) at the Testing and Quality Control Laboratory of the Universidad Católica de Santa María de Arequipa, Peru, which is an accredited laboratory in water analysis taking into account a validation process that included the evaluation of linearity, precision, accuracy, and sensitivity parameters.

Equation (1) was used to calculate the accumulation of metals in *C. album*.

(1) $$\begin{array}{c} \text{Bioaccumulation }\left(\text{mg}/\text{kg}\right)=\frac{C\times V}{w}, \end{array}$$

where “*C*” corresponds to the concentration of each metal in mg/L determined by ICP-OES, “*V*” is the volume of the flask (0.025 L) where the solution obtained after digestion of the plant material was made up, “*w*” corresponds to the weight in grams of digested plant material, in kg.

### 2.7. Quantification of Cd, Pb, and Hg by ICP-OES

Table 2 shows the parameters of the validation of the method for the quantification of Cd, Pb, and Hg by ICP with an (ICP-OES) where the results indicate that the method is linear (*R*^2^ > 0.995), precise (RSD < 2.0%), and accurate (90% < %R < 110%) with limits of detection and quantification suitable for the present study. These results were provided by the Testing and Quality Control Laboratory of the Universidad Católica de Santa María in Arequipa, Peru.

Another study based on the same technique was able to validate the method that was used to determine Hg in soil and plants, with a %R value of 100% [55]. In another study they were able to determine %R as 90%–105% for Cd and 96%–107% for Pb [56]. The results showed that these methods were appropriate for the quantification of the metals under study. In the present investigation it was also possible to obtain adequate %R to guarantee the reliability of the results.

## 3. Results and Discussion

### 3.1. Adaptation of *Chenopodium album* L. to Hoagland's Solution

**Figure 1:**
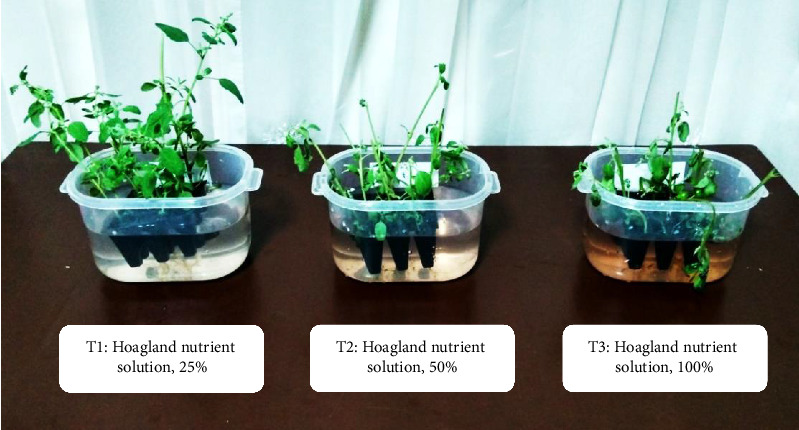
Development of *Chenopodium album* in different concentrations of 25%, 50%, and 100% Hoagland's nutrient solution.

For the study of *C. album* adaptation to HNS, three treatments were tested using HNS concentrations of 25% (T1), 50% (T2), and 100% (T3).  Figure 1 shows the *C. album* seedlings in treatments T1, T2, and T3. Treatment T1 showed better results after 15 days, observing plant growth with complete structure without relevant changes. On the other hand, in the T2 treatment, the beginnings of chlorosis were observed at 5 days of treatment, and wilting at 15 days. In the T3 treatment, seedlings showed wilting in the lower part of the stem and senescence at 6 days, with root necrosis. Therefore, the seedlings were adapted to 25% of HNS for subsequent experiments.

In another study, the effect of the nutrient solution on the growth of *Eruca sativa* was determined, where it was found that the lower the concentration of the nutrient solution, the lower the development of this species, however, this deficit in the development was corrected by enriching the nutrient solution with humic acids [57]. On the other hand, in another investigation, they worked with different concentrations of HNS of 0%, 25%, 50%, and 75%, finding that Hoagland at 75% significantly increased the growth and yield of bean cultivars [58]. On the other hand, in the present investigation it was possible to demonstrate that *C. album* can easily adapt to lower concentrations of nutrient solution without the need to enrich the nutrient solution (25%).

### 3.2. Phytotoxicity Test

The phytotoxicity and bioaccumulation tests were carried out to determine the appropriate concentration of each metal for the bioaccumulation test, which corresponds to the maximum concentration of the metal that the plant can tolerate and can accumulate the greatest amount of metal in its interior.

Table 3 shows the toxicity classification after exposure of *C. album* to different concentrations of Cd, Pb, and Hg for 19 days. In the blank, where there is an absence of metal contamination, it was found that during the 19 days, there were no symptoms of toxicity (G1). About the Cd toxicity study, it was observed that at a concentration of 5 mg/L *C. album* showed outbreaks of chlorosis at 10 days (G2), and at 13–19 days, beginnings of senescence in the lower third, and middle third with chlorosis and wrinkling of the leaves (G4), on the other hand, with a concentration of 10 mg/L, from 10 to 16 days, the plants showed slight chlorosis and continuous senescence, ending at 19 days with senescence up to the intermediate part (G5). In the 15 mg/L Cd solution, symptoms were detected after 4 days (G2), after 3 days the lower third presented chlorosis and leaf wrinkling (G4), after 13 days the lower and middle senescence began, and finally the upper third with leaf abscission (G5-G6).

Regarding Pb toxicity (Table 3), concentrations of 5, 10, and 15 mg/L were also evaluated. It was found that at a concentration of 5 mg/L, no symptoms of toxicity were observed, demonstrating tolerance to this concentration. With the concentration of 10 mg/L, at 13 days they began to show slight changes (G2), presenting small areas with chlorosis in the lower third and little wrinkling of the leaves at 19 days (G3). At the 15 mg/L concentration, at 10 days, the beginning of chlorosis was observed (G2), and from 13 to 19 days, progressive wilting was observed, ending in partial senescence (G4-G5).

In the Hg toxicity study, concentrations of 1, 5, and 10 mg/L were evaluated (Table 3). For the 1 mg/L concentration, slight changes were evidenced as slight chlorosis at 16 and 19 days (G2). With the 5 mg/L treatment, chlorosis symptoms began at 13 days (G3), continuing until the senescence of the lower third of the stem and leaf wrinkling (G3 and G4). With the 15 mg/L treatment, chlorosis began at 4 days (G3), followed by the lower, middle, and beginning of senescence at 10 days (G4), and finally at 16 days showed complete senescence and necrosis in the lower part (G6). Toxicity studies determined that the higher the concentration of Cd, Pb, and Hg, the more *C. album* seedlings showed early symptoms of foliar and stem toxicity.

At the end of the treatment, the concentration of Cd, Pb, and Hg accumulated in *C. album* was analyzed (Table 4). The maximum concentration of accumulated Cd was presented in the root part of *C. album* with 4100 ± 500 mg/kg in the treatment where the initial concentration was 15 mg/L of Cd. Also, the maximum concentration of accumulated Cd in the aerial part with 18 ± 5 mg/kg occurred in the treatment where 10 mg/L of Cd was used. Thus, it was determined that the appropriate concentration for the Cd bioaccumulation test would be 10 mg/L, since, on the toxicity scale, it showed greater resistance during more days of treatment (Table 3). The highest Pb accumulation occurred in the root part with 5800 ± 500 mg/kg where the initial concentration was 15 mg/L of Pb, as well as in the aerial part, with 21 ± 5 mg/kg (Table 4). When contrasted with the toxicity scale, it was determined that the concentration of 10 mg/L would be the most appropriate due to the resistance shown by *C. album* to this concentration of Pb (Table 3). Regarding Hg, a maximum accumulation of 221 ± 33 mg/kg of Hg was found in the aerial part at an initial concentration of 5 mg/L Hg. The highest Hg accumulation in the aerial part was 8400 ± 480 mg/kg at an initial concentration of 10 mg/L Hg (Table 4). Assessing the toxicity scale, it was determined that the appropriate concentration to carry out the final bioaccumulation test would be a concentration of 5 mg/L since the highest Hg accumulation and the highest Hg resistance were obtained (Table 3).

Some plant species are able to adapt and withstand extreme environmental conditions contaminated with heavy metals [52], as is the case of *C. album* which is considered a heavy metal accumulator species in studies on soils and sewage sludge [51]. However, there was no data on the phytoremediation capacity of *C. album* in water, which was one of the findings of the present research, demonstrating that this species can adapt its growth in hydroponic media and tolerate concentrations of Cd, Pb, and Hg.

In another study it was demonstrated that *C. album* in specific MS medium for plate culture was able to tolerate concentrations of up to 50 mg/L without showing variations in its size and weight with a low malondialdehyde (MDA) production (≥ 600 mg/L) which is formed when the plant is under stress. However, *C. album* had low MDA concentrations which could be attributed to the fact that *C. album* develops antioxidant enzyme activity to reduce H_2_O_2_ levels and thus minimize damage to the membrane which would explain its ability to adapt to the new conditions [49].

### 3.3. Bioaccumulation Assay

#### 3.3.1. Bioaccumulation of Cd, Pb, and Hg in Monometallic Aqueous Systems

**Figure 2:**
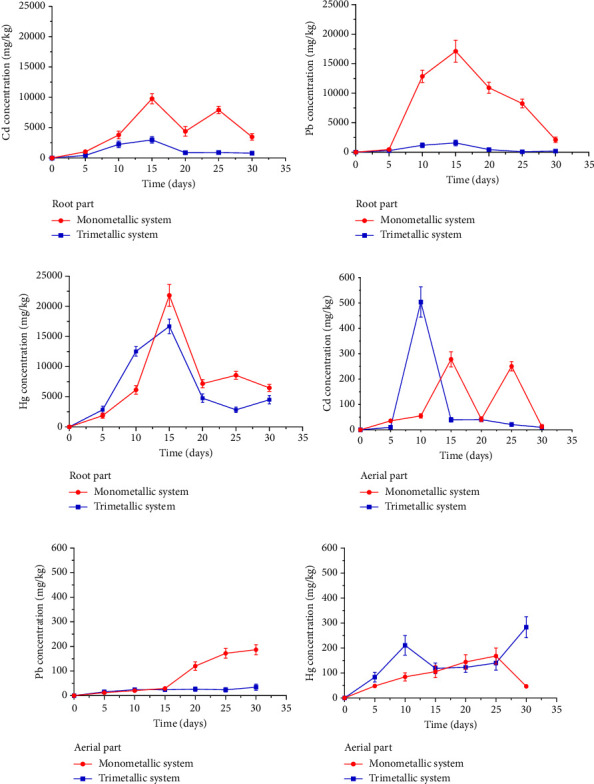
Bioaccumulation of Cd (a), Pb (b), and Hg (c) in root parts and Cd (d), Pb (e), and Hg (f) in aerial parts of *Chenopodium album* in monometallic (red line) and trimetallic (blue line) aqueous systems.

**Figure 3:**
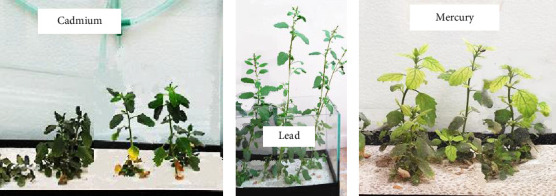
*Chenopodium album* seedlings after the bioaccumulation study of (a) Cd, (b) Pb, and (c) Hg.

For the Cd, Pb, and Hg bioaccumulation assay, 7 cm seedlings were used. The results are presented graphically in Figure 2 (red line). Regarding the bioaccumulation of Cd in the root part, the accumulation of this metal is evident from 5 days with a concentration of 1030 ± 290 mg/kg, reaching a maximum accumulation of 9770 ± 850 mg/kg at 15 days, then an irregular behavior is observed after 20 days (Figure 2(a)). In the aerial part, accumulation of this metal is also observed from day 5, reaching a maximum accumulation of 280 ± 30 mg/kg at 15 days, and after this, the same irregular behavior as in the roots is observed, as well as a decline in accumulation on day 30 (Figure 2(d). This would be due to a process of metal release by the plant through an excretion mechanism [59]. The root part showed a greater capacity for Cd accumulation than the aerial part, which demonstrates that *C. album* would use rhizofiltration and phytoextraction processes [60] to decontaminate water with Cd.  Figure 3(a) shows *C. album* seedlings after 30 days in water contaminated with Cd, these seedlings managed to grow to a size of 10 cm in height, with this it could be said that the presence of Cd in the hydroponic medium reduced the normal growth of *C. album* since the seedlings of the control group (without heavy metal contamination) managed to grow to a size of approximately 65 cm.

Regarding Pb bioaccumulation, the root part presented an accumulation of 470 ± 60 mg/kg at 5 days, increasing at 10 days and reaching a maximum accumulation of 17,000 ± 1900 mg/kg at 15 days of treatment; a decay was evidenced at 20 days due to a possible process of release by saturation of the metal in the root (Figure 2(b)). On the other hand, in the aerial part, an increasing accumulation was observed from day 5, accumulating the highest amount of Pb at 190 ± 20 mg/kg at 30 days (Figure 2(e)). At this point, the translocation of Pb is evidenced, which would be related to plant growth, as was verified by [61] in species such as *Sedum spectabile* and *Urtica dioica*. There would be a greater accumulation of Pb in the root part compared to the aerial part due to the slow translocation of this element from the root to the aerial part; this is explained by Manousaki et al. [62] who evaluated this phenomenon in *Tamarix smyrnensis* plants. Therefore, it could be said that *C. album* uses rhizofiltration and phytoextraction processes for decontamination of Pb-containing waters. Considering that the maximum accumulation was obtained in the root part at 15 days and in the aerial part at 30 days, it could be said that this is due to translocation to the leaves; however, the accumulation in the root part was much greater, considering 15 days for the maximum absorption of Pb in the plant. Regarding the growth of *C. album* seedlings in the presence of Pb, it was found that after 30 days they were able to grow up to 40 cm in height (Figure 3(b)), with this it could be said that *C. album* would tolerate the presence of Pb better than Cd (Figure 3(c)), however, Pb would also be limiting the growth of *C. album* in comparison to the control group.

Regarding the bioaccumulation of Hg in the root zone, it was observed that there was a considerable accumulation of 1900 ± 400 mg/kg 5 days after the beginning of the treatment and after 15 days the highest accumulation occurred with a concentration of 21,800 ± 1800 mg/kg, beginning a decay after 20 days (Figure 2(c)). On the other hand, in the aerial part, an increasing accumulation was observed, also from day 5, accumulating the highest amount of Hg 170 ± 30 mg/kg at 25 days, showing decay at 30 days (Figure 2(f)), this could be due to the growth of aerial biomass. On the other hand, the root part showed a greater capacity for Hg accumulation than the aerial part, so it could be said that *C. album* would use the process of rhizofiltration and phytoextraction to remove Hg from contaminated water [60]. At 15 days the maximum accumulation was obtained in the root part, and the aerial part at 25 days, this time difference was due to the translocation of the metal to the aerial part, however, the accumulation in the root part was greater, thus considering 15 days for the maximum absorption of Hg. Figure 3(c) shows that *C. album* seedlings at 30 days managed to grow up to 16 cm in the presence of Hg, also a yellow coloration of the leaves characteristic of chlorosis is observed, also, it can be said that Hg would limit the growth of *C. album* since the growth is much lower compared to the control group (65 cm).

#### 3.3.2. Bioaccumulation of Cd, Pb, and Hg in Trimetallic Systems

The bioaccumulation results of Cd, Pb, and Hg in trimetallic aqueous systems are presented in Figure 2 (blue line). Regarding the bioaccumulation of Cd in the trimetallic solutions, it is observed that in the root part the bioaccumulation increases, with a maximum accumulation of 3000 ± 550 mg/kg at 15 days and decay at 20 days, maintaining the same irregular behavior until day 30 (Figure 2(a)). The aerial part presented a maximum absorption of 500 ± 60 mg/kg at 10 days, with a decreasing behavior starting at 15 days (Figure 2(d)) which could be due to an increase in plant biomass. The time of maximum accumulation in the root part was at 15 days and in the aerial part at 10 days; this difference was due to the time of translocation; however, the accumulation in the root part is higher than in the monometallic aqueous system, the maximum absorption was considered at 15 days. In contrast to the root part, the seedlings of the trimetallic solution treatment showed a greater accumulation of Cd in the aerial part of the plant. Thus, *C. album*, in the presence of other metals, increases its translocation and transport capacity to the leaves, accumulating more Cd in the aerial part of the plant. The highest accumulation of Cd occurred at 15 days. Then, an analysis of the whole plant was made, adding the amounts accumulated in the root and aerial parts to determine the influence of metals on accumulation. The accumulation capacity of Cd by *C. album*, in the presence of Pb and Hg, was 30%.

Regarding Pb in the root part, an irregular behavior was observed, with a maximum accumulation of 1500 ± 500 mg/kg at 15 days and the same decay as the monometallic aqueous system at 20 days (Figure 2(b)). On the other hand, the aerial part presented an accumulation of 25 ± 6 mg/kg at 10 days, maintained until 25 days, increasing to a maximum accumulation of 34 ± 13 mg/kg at 30 days (Figure 2(e)). The bioaccumulation of Pb in trimetallic solutions showed a concentration of Pb approximately 10 times lower than in monometallic solutions in both the aerial and root parts, demonstrating that there is interference from other metals in the capacity of Pb accumulation in *C. album*. The highest Pb accumulation occurred at 15 days. The Pb accumulation capacity of *C. album*, in the presence of other contaminating metals such as Hg and Cd, was 9%.

About Hg bioaccumulation in trimetallic solutions, it is observed that the root part achieves the maximum accumulation of 16,700 ± 1200 mg/kg at 15 days and the same decay behavior as the monometallic solutions at 20 days (Figure 2(c)). In the aerial part, an irregular behavior was observed, presenting a maximum accumulation of 280 ± 40 mg/kg at 30 days (Figure 2(f)). In trimetallic aqueous systems, the time of maximum Hg accumulation in the root part was at 15 days and in the aerial part at 30 days, this difference was due to the time of translocation of the metal to the aerial part, however, the accumulation in the root part was greater, as in the monometallic aqueous system, the maximum accumulation was considered to be at 15 days. During the 30 days of treatment, the seedlings of the trimetallic aqueous systems showed greater accumulation of Hg in the aerial part denoting the increase in translocation, but much less than in the root part. The highest Hg accumulation occurred at 15 days. The Hg accumulation capacity of *C. album* in the presence of Cd and Pb was 77%.

Different phytoremediation studies in soils where *C. album* has been used have shown that this species is effective in accumulating heavy metals. In a study of phytoremediation of Pb in soils using *C. album*, accumulated lead content was found in lower concentration in the aerial part than in the subway part (root) [63]. Another study evaluated the ability of *C. album* to accumulate different heavy metals (Cd, As, Pb, Cu, Zn, and Cr) in soils simultaneously and found that this species tends to accumulate only one metal [48]. Another study conducted in soil samples confirmed this finding, where it was found that *C. album* was able to accumulate the highest concentration of Cd in the roots [38]. Main accumulation mechanisms of *C. album* in soil were considered as phytostabilization and phytoextraction of heavy metals [64]. Studies were also conducted in soils contaminated with wastewater or effluents, proved the ability of *C. album* to adapt to more extreme conditions. This is demonstrated in a study in soils contaminated with tannery sludge where they found that *C. album* was able to accumulate heavy metals in the following order Fe > Mn > Zn > Cr > Cu > Pb > Ni > Cd and the mechanism of accumulation was phytoextraction [47]. On the other hand, another study showed that *C. album* was able to adapt and grow in sludge from pulp and paper mill effluents, accumulating Mn, Pb, Cd, Zn, Cr, Fe, Cu, Ni, As, and Hg in roots, shoots, and leaves through bioconcentration and translocation [50].

In order to cope with different types of metals, plants possess defense strategies related to cellular free metal content (e.g., metal exclusion, cell wall binding, chelation and sequestration, and regulation of cellular responses) [65, 66]. On the other hand, GSH is known to be a key component in metal removal due to the high affinity of metals to their thiol group (−SH) and as a precursor of phytochelatins, in addition to metal homeostasis, plants possess an antioxidant defense system well equipped to handle the oxidative challenge imposed by metal [67, 68]. The cysteine residue in GSH makes it an important antioxidant that, in addition to its primary antioxidant capabilities, acts as a substrate for the regeneration of other essential antioxidants [69, 70]. Thus, GSH plays a role in both metal homeostasis and antioxidant defense, both of which influence free reduced GSH levels [70]. In the case of *C. album* in a study conducted in soils contaminated with Cd, Cu, and Pb, it was found that the concentration of heavy metals correlated with an increase in GSH and plant cysteine, in addition, a significant increase in the concentration of short-chain phytochelatins was observed [71].

In the present research it was found that *C. album* can adapt to hydroponic environments, which would be important for the food industry in countries where this species is included in the diet. However, its high ability to adapt and colonize contaminated sites [40]; it can represent a constant health risk and can be a pathway for the accumulation of heavy metals to human beings, which could lead to alterations in many tissues and organs [72]. For this reason, it is recommended that soil and water where they are grown be analyzed to avoid repercussions on the health of consumers.

## 4. Conclusions

*C. album* was shown to be a species with phytoremediation potential for Cd, Pb, and Hg in monometallic aqueous solutions. In trimetallic systems, it was demonstrated that *C. album* accumulates Cd, Pb, and Hg simultaneously, being the root accumulating a higher concentration of each metal compared to aerial parts. It was possible to demonstrate that *C. album* shows phytoextraction and rhizofiltration mechanisms of Cd, Pb, and Hg in both monometallic and trimetallic aqueous systems. The results of the current study may aid in the development of the food industry for the production of *C. album* in hydroponic systems. Our results also proved the capacity of *C. album* to remediate river water, irrigation water, wastewater, effluents, and mining tailings contaminated with Pb, Cd, and Hg.

## Figures and Tables

**Table 1:** Scale to evaluate damage and/or toxicity degrees in aerial part (AP) and root part (RP) [53].

Toxicity levels	Description
Grade 1 (G1)	RP: no symptoms were observed
	AP: no symptoms were observed

Grade 2 (G2)	RP: no symptoms were observed
	AP: lower third with chlorotic areas, middle and upper third no symptoms were observed

Grade 3 (G3)	RP: no symptoms were observed
	AP: lower third with chlorosis and/or leaf wrinkling, middle third with chlorotic areas

Grade 4 (G4)	RP: no symptoms were observed
	AP: lower third with beginning of senescence, middle third with chlorosis and wrinkling, upper third without symptoms

Grade 5 (G5)	RP: no symptoms were observed
	AP: lower third with senescence in its entirety and leaf abscission. Middle third with senescence and formation of new axillary buds, upper third with buds

Grade 6 (G6)	RP: no symptoms were observed
	AP: lower third with complete senescence and leaf abscission, middle third with senescence and formation of new axillary buds, upper third with complete senescence and leaf abscission

**Table 2:** Values of validation parameters for inductively coupled plasma quantification methods of Cd, Pb, and Hg (ICP-OES).

Element	a	b	*R* ^2^	RSD (%)	%R	LOD (µg/L)	LOQ (µg/L)
Cd	−292.5	85,170	0.9999	0.33	101.21	0.0003	0.0008
Pb	−28.5	5229	0.9999	1.16	98.99	0.0146	0.0442
Hg	−20.5	23,410	0.9997	1.23	97.54	0.0001	0.0002

*Note:* a = intercept, b = slope, *R*^2^ = coefficient of determination.

Abbreviations: %R = percentage of recovery, LOD = limit of detection, LOQ = limit of quantification, RSD = relative standard deviation.

**Table 3:** Results of the toxicity study according to toxicity scale at different concentrations of Cd, Pb, and Hg in *Chenopodium album* for 19 days.

Metal	Treatment (mg/L)	Days							
		0	1	4	7	10	13	16	19
Cd	0	G1	G1	G1	G1	G1	G1	G1	G1
	5	G1	G1	G1	G1	G2	G4	G4	G4
	10	G1	G1	G1	G1	G2	G3	G4	G5
	15	G1	G1	G2	G3	G4	G5	G5	G6

Pb	0	G1	G1	G1	G1	G1	G1	G1	G1
	5	G1	G1	G1	G1	G1	G1	G1	G1
	10	G1	G1	G1	G1	G1	G2	G2	G3
	15	G1	G1	G1	G1	G2	G4	G5	G5

Hg	0	G1	G1	G1	G1	G1	G1	G1	G1
	1	G1	G1	G1	G1	G1	G1	G2	G2
	5	G1	G1	G1	G1	G1	G2	G3	G4
	10	G1	G1	G3	G3	G4	G5	G6	G6

**Table 4:** Bioaccumulation of Cd, Pb, and Hg in the root and aerial parts of *Chenopodium album* after the toxicity tests.

Metal	Concentration in water (mg/L)	Concentration in *C. album* (mg/kg)	
		Root part	Aerial part
Cd	0	ND	ND
	5	1500 ± 300	17 ± 3
	10	3400 ± 300	18 ± 5
	15	4100 ± 500	11 ± 3

Pb	0	ND	ND
	5	1100 ± 200	15 ± 3
	10	1800 ± 250	15 ± 3
	15	5800 ± 500	21 ± 5

Hg	0	ND	ND
	1	42 ± 9	32 ± 5
	5	2700 ± 200	221 ± 33
	10	8400 ± 480	140 ± 25

Abbreviation: ND, not detectable.

## Data Availability

The data that support the findings of this study are available from the corresponding author upon reasonable request.

## References

[1] Sharma S., Bhattacharya A. (2017). Drinking Water Contamination and Treatment Techniques. *Applied Water Science*.

[2] Ghadouani A., Coggins L. X. (2011). Science, Technology and Policy for Water Pollution Control at the Watershed Scale: Current Issues and Future Challenges. *Physics and Chemistry of the Earth, Parts A/B/C*.

[3] Abdul Maulud K. N., Fitri A., Wan Mohtar W. H. M., Wan Mohd Jaafar W. S., Zuhairi N. Z., Kamarudin M. K. A. (2021). A Study of Spatial and Water Quality Index During Dry and Rainy Seasons at Kelantan River Basin, Peninsular Malaysia. *Arabian Journal of Geosciences*.

[4] Farré M., Martínez E., Barceló D. (2007). Validation of Interlaboratory Studies on Toxicity in Water Samples. *TrAC, Trends in Analytical Chemistry*.

[5] Arenas-Sánchez A., Dolédec S., Vighi M., Rico A. (2021). Effects of Anthropogenic Pollution and Hydrological Variation on Macroinvertebrates in Mediterranean Rivers: A Case-Study in the Upper Tagus River Basin (Spain). *Science of the Total Environment*.

[6] Karri R. R., Ravindran G., Dehghani M. H., Karri R. R., Ravindran G., Dehghani M. H. (2021). Chapter 1-Wastewater—Sources, Toxicity, and Their Consequences to Human Health. *Soft Computing Techniques in Solid Waste and Wastewater Management*.

[7] Ali H., Khan E., Ilahi I. (2019). Environmental Chemistry and Ecotoxicology of Hazardous Heavy Metals: Environmental Persistence, Toxicity, and Bioaccumulation. *Journal of Chemistry*.

[8] Wasewar K. L., Singh S., Kansal S. K., Devi P., Singh P., Kansal S. K. (2020). Chapter 13-Process Intensification of Treatment of Inorganic Water Pollutants. *Inorganic Pollutants in Water*.

[9] Liang Y., Pan D., Wang C., Lu Y., Fan X. (2022). Distribution and Ecological Health Risk Assessment of Dissolved Trace Metals in Surface and Bottom Seawater of Yantai Offshore, China. *Frontiers in Marine Science*.

[10] Haider F. U., Liqun C., Coulter J. A. (2021). Cadmium Toxicity in Plants: Impacts and Remediation Strategies. *Ecotoxicology and Environmental Safety*.

[11] Mahmood Q., Asif M., Shaheen S., Hayat M. T., Ali S., Hasanuzzaman M., Prasad M. N. V., Fujita M. (2019). Chapter 6-Cadmium Contamination in Water and Soil. *Cadmium Toxicity and Tolerance in Plants*.

[12] Ellen T. P., Costa M., McQueen C. A. (2010). 14.08-Carcinogenic Inorganic Chemicals. *Comprehensive Toxicology*.

[13] Raj J., Raina A., Dogra T. D., Dogra T. D. (2013). Direct Determination of Zinc, Cadmium, Lead, Copper Metal in Tap Water of Delhi (India) by Anodic Stripping Voltammetry Technique. *E3S Web of Conferences*.

[14] Das S., Sultana K. W., Ndhlala A. R., Mondal M., Chandra I. (2023). Heavy Metal Pollution in the Environment and Its Impact on Health: Exploring Green Technology for Remediation. *Environmental Health Insights*.

[15] Mushak P. (2011). A Brief Early History of Lead as an Evolving Global Pollutant and Toxicant. *Trace Metals and Other Contaminants in the Environment*.

[16] Wani A. L., Ara A., Usmani J. A. (2015). Lead Toxicity: A Review. *Interdisciplinary Toxicology*.

[17] Taux K., Kraus T., Kaifie A. (2022). Mercury Exposure and its Health Effects in Workers in the Artisanal and Small-Scale Gold Mining (ASGM) Sector—A Systematic Review. *International Journal of Environmental Research and Public Health*.

[18] Kocman D., Wilson S. J., Amos H. M. (2017). Toward an Assessment of the Global Inventory of Present-Day Mercury Releases to Freshwater Environments. *International Journal of Environmental Research and Public Health*.

[19] Budnik L. T., Casteleyn L. (2019). Mercury Pollution in Modern Times and Its Socio-Medical Consequences. *Science of the Total Environment*.

[20] Bernhoft R. A. (2012). Mercury Toxicity and Treatment: A Review of the Literature. *Journal of Environmental and Public Health*.

[21] Yi Y. S., Han Y., Hur S. D. (2021). Reductive Adsorption of Atmospheric Oxidized Mercury on Ice: Insights from Density Functional Calculations. *ACS Earth and Space Chemistry*.

[22] Balali-Mood M., Naseri K., Tahergorabi Z., Khazdair M. R., Sadeghi M. (2021). Toxic Mechanisms of Five Heavy Metals: Mercury, Lead, Chromium, Cadmium, and Arsenic. *Frontiers in Pharmacology*.

[23] Posin S. L., Kong E. L., Sharma S. (2021). *Mercury Toxicity*.

[24] Gao Z., Wu N., Du X., Li H., Mei X., Song Y. (2022). Toxic Nephropathy Secondary to Chronic Mercury Poisoning: Clinical Characteristics and Outcomes. *Kidney International Reports*.

[25] Sarkar O., Dey K. K., Islam S., Chattopadhyay A., Patel V. B., Preedy V. R., Rajendram R. (2023). Lead and Aquatic Ecosystems, Biomarkers, and Implications for Humankind. *Biomarkers in Toxicology*.

[26] Lee J.-W., Choi H., Hwang U.-K. (2019). Toxic Effects of Lead Exposure on Bioaccumulation, Oxidative Stress, Neurotoxicity, and Immune Responses in Fish: A Review. *Environmental Toxicology and Pharmacology*.

[27] Liu Y., Chen Q., Li Y., Bi L., Jin L., Peng R. (2022). Toxic Effects of Cadmium on Fish. *Toxics*.

[28] Lee J.-W., Jo A.-H., Lee D.-C., Choi C. Y., Kang J.-C., Kim J.-H. (2023). Review of Cadmium Toxicity Effects on Fish: Oxidative Stress and Immune Responses. *Environmental Research*.

[29] Zheng N., Wang S., Dong W. (2019). The Toxicological Effects of Mercury Exposure in Marine Fish. *Bulletin of Environmental Contamination and Toxicology*.

[30] Briffa J., Sinagra E., Blundell R. (2020). Heavy Metal Pollution in the Environment and Their Toxicological Effects on Humans. *Heliyon*.

[31] Razzak S. A., Faruque M. O., Alsheikh Z. (2022). A Comprehensive Review on Conventional and Biological-Driven Heavy Metals Removal from Industrial Wastewater. *Environmental Advances*.

[32] Vocciante M., Caretta A., Bua L. (2019). Enhancements in Phytoremediation Technology: Environmental Assessment Including Different Options of Biomass Disposal and Comparison with a Consolidated Approach. *Journal of Environmental Management*.

[33] Kumar S., Thakur N., Singh A. K., Gudade B. A., Ghimire D., Das S., Kumar V., Shah M. P., Shahi S. K. (2022). Microbes-Assisted Phytoremediation of Contaminated Environment: Global Status, Progress, Challenges, and Future Prospects. *Phytoremediation Technology for the Removal of Heavy Metals and Other Contaminants from Soil and Water*.

[34] Poma Llantoy V. R., Valderrama Negrón A. C. (2014). Study of Physicochemical Parameters for Cadmium (ii) and Mercury (Ii) Phytoremediation Using the Specie *Eichhornia Crassipes* (Water Hyacinth). *Journal of the Chemical Society of Peru*.

[35] Bamagoos A. A., Alharby H. F., Abbas G. (2022). Differential Uptake and Translocation of Cadmium and Lead by Quinoa: A Multivariate Comparison of Physiological and Oxidative Stress Responses. *Toxics*.

[36] Eapen S., Suseelan K. N., Tivarekar S., Kotwal S. A., Mitra R. (2003). Potential for Rhizofiltration of Uranium Using Hairy Root Cultures of *Brassica Juncea* and *Chenopodium Amaranticolor*. *Environmental Research*.

[37] Haseeb M., Iqbal S., Hafeez M. B. (2023). Characterizing of Heavy Metal Accumulation, Translocation and Yield Response Traits of *Chenopodium Quinoa*. *Journal of Agriculture and Food Research*.

[38] Naz R., Khan M. S., Hafeez A. (2022). Assessment of Phytoremediation Potential of Native Plant Species Naturally Growing in a Heavy Metal-Polluted Industrial Soils. *Brazilian Journal of Biology*.

[39] Yuan Y., Yu S., Bañuelos G. S., He Y. (2016). Accumulation of Cr, Cd, Pb, Cu, and Zn by Plants in Tanning Sludge Storage Sites: Opportunities for Contamination Bioindication and Phytoremediation. *Environmental Science & Pollution Research*.

[40] Walker D. J., Clemente R., Bernal M. P. (2004). Contrasting Effects of Manure and Compost on Soil pH, Heavy Metal Availability and Growth of *Chenopodium album* L. In a Soil Contaminated by Pyritic Mine Waste. *Chemosphere*.

[41] Rashid Shomali R. S., Khodaverdiloo H., Samadi A. (2012). Accumulation and Tolerance of Soil Cadmium Contamination by Millet (Pennisetum Glaucum), Lambsquarter (Chenopodium Album). *Flix Weed (Descurainia Sophi) and Purslane (Portulaca Oleracea)*.

[42] Zulfiqar S., Wahid A., Farooq M., Maqbool N., Arfan M. (2012). Phytoremediation of Soil Cadmium Using Chenopodium Species. *Pakistan Journal of Agricultural Sciences*.

[43] Ebrahimi M. (2016). Enhanced Phytoremediation Capacity of *Chenopodium album* L. Grown on Pb-Contaminated Soils Using EDTA and Reduction of Leaching Risk. *Soil and Sediment Contamination: International Journal*.

[44] Mohan A., Kaur R., Girdhar M. (2019). Analysis of Ability of *Chenopodium album* for Remediation of Heavy Metal Degraded Soil. *Research Journal of Pharmacy and Technology*.

[45] Tőzsér D., Tóthmérész B., Harangi S. (2019). Remediation Potential of Early Successional Pioneer Species *Chenopodium album* and Tripleurospermum Inodorum. *Nature Conservation*.

[46] Moogouei R., Borghei M., Arjmandi R. (2011). Phytoremediation of Stable Cs from Solutions by Calendula Alata, Amaranthus Chlorostachys and *Chenopodium album*. *Ecotoxicology and Environmental Safety*.

[47] Gupta A. K., Sinha S. (2007). Phytoextraction Capacity of the *Chenopodium album* L. Grown on Soil Amended with Tannery Sludge. *Bioresource Technology*.

[48] Wang Q., Huang S., Jiang R. (2023). Phytoremediation Strategies for Heavy Metal-Contaminated Soil by Selecting Native Plants Near Mining Areas in Inner Mongolia. *Environmental Science & Pollution Research*.

[49] Hu R., Sun K., Su X., Pan Y., Zhang Y., Wang X. (2012). Physiological Responses and Tolerance Mechanisms to Pb in Two Xerophils: Salsola passerina Bunge and *Chenopodium album* L. *Journal of Hazardous Materials*.

[50] Sharma P., Tripathi S., Chandra R. (2020). Phytoremediation Potential of Heavy Metal Accumulator Plants for Waste Management in the Pulp and Paper Industry. *Heliyon*.

[51] González-Ramírez L. R., Alaçam D., Akpinar A. (2022). A Mathematical Model of *Chenopodium album* L. Dynamics Under Copper-Induced Stress. *Ecological Modelling*.

[52] Phang L.-Y., Mohammadi M., Mingyuan L. (2023). Underutilised Plants as Potential Phytoremediators for Inorganic Pollutants Decontamination. *Water, Air, and Soil Pollution*.

[53] Veitía N., Dita M. A., García L. (2001). Use of Tissue Culture and In Vitro Mutagenesis for the Improvement of Resistance to Alternaria solani in Potato Var. ¨Desirée. *Vegetal Biotechnology*.

[54] Han F. X., Patterson W. D., Xia Y., Sridhar B. B. M., Su Y. (2006). Rapid Determination of Mercury in Plant and Soil Samples Using Inductively Coupled Plasma Atomic Emission Spectroscopy, a Comparative Study. *Water, Air, and Soil Pollution*.

[55] Hellings J., Adeloju S. B., Verheyen T. V. (2013). Rapid Determination of Ultra-Trace Concentrations of Mercury in Plants and Soils by Cold Vapour Inductively Coupled Plasma-Optical Emission Spectrometry. *Microchemical Journal*.

[56] Zhang N., Shen K., Yang X. (2018). Simultaneous Determination of Arsenic, Cadmium and Lead in Plant Foods by ICP-MS Combined With Automated Focused Infrared Ashing and Cold Trap. *Food Chemistry*.

[57] Sarabi B. (2024). Effect of Nutrient Solution Concentrations and Irrigation Levels Combined With Humic Acid on Physiological and Quality Characteristics of Rocket Crop (*Eruca sativa* (mill.) Thell.). *Arid Land Research and Management*.

[58] Waheed H., Javaid M. M., Shahid A., Ali H. H., Nargis J., Mehmood A. (2019). Impact of Foliar-Applied Hoagland’s Nutrient Solution on Growth and Yield of Mash Bean (*Vigna mungo* L.) under Different Growth Stages. *Journal of Plant Nutrition*.

[59] Uraguchi S., Mori S., Kuramata M., Kawasaki A., Arao T., Ishikawa S. (2009). Root-to-Shoot Cd Translocation via the Xylem Is the Major Process Determining Shoot and Grain Cadmium Accumulation in Rice. *Journal of Experimental Botany*.

[60] Yadav B. K., Siebel M. A., van Bruggen J. J. A. (2011). Rhizofiltration of a Heavy Metal (Lead) Containing Wastewater Using the Wetland Plant Carex Pendula. *Clean: Soil, Air, Water*.

[61] Grubor M. (2008). Lead Uptake, Tolerance, and Accumulation Exhibited by the Plants Urtica Dioica and Sedum Spectabile in Contaminated Soil Without Additives. *Archives of Biological Sciences*.

[62] Manousaki E., Kadukova J., Papadantonakis N., Kalogerakis N. (2008). Phytoextraction and Phytoexcretion of Cd by the Leaves of Tamarix Smyrnensis Growing on Contaminated Non-saline and Saline Soils. *Environmental Research*.

[63] Bai Y., Xie Y. (2011). Effects of Exogenous Lead on the Growth and Lead Accumulation Characteristics of Roadside Dominant Herbaceous Plants in Shanxi Province. *Yingyong Shengtai Xuebao*.

[64] Irshad M., Ruqia B., Hussain Z. (2015). Phytoaccumulation of Heavy Metals in Natural Vegetation at the Municipal Wastewater Site in Abbottabad, Pakistan. *International Journal of Phytoremediation*.

[65] Jozefczak M., Remans T., Vangronsveld J., Cuypers A. (2012). Glutathione Is a Key Player in Metal-Induced Oxidative Stress Defenses. *International Journal of Molecular Sciences*.

[66] Kushwaha A., Rani R., Kumar S., Gautam A. (2016). Heavy Metal Detoxification and Tolerance Mechanisms in Plants: Implications for Phytoremediation. *Environmental Review*.

[67] Cuypers A., Plusquin M., Remans T. (2010). Cadmium Stress: an Oxidative Challenge. *Biometals*.

[68] Lwalaba J. L. W., Louis L. T., Zvobgo G. (2020). Physiological and Molecular Mechanisms of Cobalt and Copper Interaction in Causing Phyto-Toxicity to Two Barley Genotypes Differing in Co Tolerance. *Ecotoxicology and Environmental Safety*.

[69] Bashandy T., Guilleminot J., Vernoux T. (2010). Interplay between the NADP-Linked Thioredoxin and Glutathione Systems in Arabidopsis Auxin Signaling. *The Plant Cell*.

[70] Averill-Bates D. A., Litwack G. (2023). The Antioxidant Glutathione. *Vitamins and Hormones*.

[71] Hunaiti A. A., Al-Oqlah A., Shannag N. M. (2007). Toward Understanding the Influence of Soil Metals and Sulfate Content on Plant Thiols. *Journal of Toxicology and Environmental Health, Part A*.

[72] Ugulu I., Khan Z. I., Rehman S. (2022). Heavy Metal Accumulation in Goosefoot (*Chenopodium album* L.) Irrigated with Wastewater. *Pakistan Journal of Analytical & Environmental Chemistry*.

